# P18 Bullous leukocytoclastic vasculitis in a patient with known undifferentiated autoimmune rheumatic disease

**DOI:** 10.1093/rap/rkad070.039

**Published:** 2023-09-27

**Authors:** Rosemary Clarke, Kazi M Fardeen, Sundeep Ghuman, Saerrah Murryam, Chris Bunker, Alexis Jones, Jessica Manson

**Affiliations:** Rheumatology, University College London Hospital, London, United Kingdom; Rheumatology, University College London Hospital, London, United Kingdom; Rheumatology, University College London Hospital, London, United Kingdom; Dermatology, University College London Hospital, London, United Kingdom; Dermatology, University College London Hospital, London, United Kingdom; Rheumatology, University College London Hospital, London, United Kingdom; Rheumatology, University College London Hospital, London, United Kingdom

## Abstract

**Introduction:**

A 55-year-old woman with undifferentiated autoimmune rheumatic disease (uAIRD) and chronic leg ulcers presented with a progressive bullous eruption on her legs. During her admission, she developed visual disturbance, hearing loss, and brain lesions were discovered on imaging. There was diagnostic uncertainty between an infective process versus an inflammatory vasculitis as the driver for her presentation. Despite an infection work-up, including multiple blood cultures and an ASOT test, no infection was identified. Ultimately, a skin biopsy showed leukocytoclastic bullous vasculitis. This case demonstrated a complex presentation of cutaneous vasculitis, where differentiation from infection was difficult, as is often the case.

**Case description:**

A 55-year-old woman presented to the emergency department, after being referred by her general practitioner, with a high CRP and a new bilateral blistering rash on her lower limbs. She had a history of recurrent leg ulcers, juvenile idiopathic arthritis (JIA), and uAIRD, with skin rash and peripheral neuropathy, and was receiving treatment with azathioprine, hydroxychloroquine and abatacept. Previous skin biopsies of the leg ulcers had shown post-inflammatory changes and non-specific ulceration. On examination, she exhibited lower limb oedema and erythema with localised blisters.

Blood tests at time of presentation showed a mild acute kidney injury (AKI), and elevated CRP (186mg/L), white cell count (WCC)(34x10^9^/L) and neutrophils (31x10^9^/L). The patient was ANA positive, anti-Ro and anti-RNP positive, ANCA positive, but MPO and PR3 negative, dsDNA negative, and C3 was low whilst C4 was normal. Given the initial impression of an infectious process, antibiotics were initiated, and a skin biopsy was performed.

The following morning the patient developed sudden-onset unilateral vision loss and auditory change. MRI head and orbits revealed multiple parenchymal brain lesions suggestive of either inflammatory or embolic aetiology, with no orbital abnormalities. The ophthalmology team suspected bilateral central retinal vein occlusion. The patient awaits formal audiometry.

Swabs of the skin lesions grew Staphylococcus aureus, Proteus mirabilis, and Corynebacteria. MRI ankle suggested deep soft tissue infection without osteomyelitis. A PET-CT did not highlight any medium/large vessel vasculitis, malignancies or abscesses.

Treatment with antibiotics did not result in any clinical improvement, or impact on inflammatory markers, with peak values of: CRP 301mg/L and WCC 52x10^9^/L. Skin histopathology revealed leukocytoclastic vasculitis, and the patient was given pulsed methylprednisolone, and cyclophosphamide. The patient then displayed a rebound flare of vasculitis, prompting the decision to give a further dose of cyclophosphamide and a cycle of rituximab.

**Discussion:**

During the early phase of the patient’s admission the diagnostic challenge revolved around the aetiology of her skin presentation, and whether the nature of her multi-systemic illness was infective or inflammatory. Initial evidence in favour of infection were high inflammatory markers, tachycardia with temperatures of 38° Celsius providing a septic picture, immunosuppressed state of the patient, MRI ankle showing fasciitis, and previous skin biopsies of the lower limbs showing no indication of vasculitis. Therefore, infection was initially believed to be the most likely cause whilst awaiting histopathology. The skin biopsy was crucial in determining the presence of vasculitis, and guiding initiation of immunosuppressant treatment.

The patient truly required a multi-specialty collaborative approach, and was reviewed by numerous specialties including dermatology, infectious diseases, microbiology, neurology, cardiology, intensive care, ophthalmology, ear nose and throat, and rheumatology.

Leukocytoclastic vasculitis can arise from various primary and secondary causes, including but not limited to ANCA vasculitis; immune complex vasculitis; systemic rheumatological diseases such as systemic lupus erythematosus (SLE), rheumatoid arthritis, and Sjogren’s syndrome; medications; infection (e.g. streptococcal); and malignancy. It is unclear as to what was the exact cause of this patient’s vasculitis. However, possibilities include involvement of the patient’s undifferentiated connective tissue disease, where progression into Sjogren’s syndrome and SLE had been queried in the past.

The management of leukocytoclastic vasculitis with systemic involvement typically entails corticosteroids with consideration of immunosuppressants, rituximab, and plasma exchange. Given the central nervous system involvement in this patient, cyclophosphamide was initiated, with rituximab as second line treatment due to the rebound of fevers and increased inflammatory markers following the initial cyclophosphamide dose.

**Key learning points:**

In retrospect, the elevated WCC and neutrophils were likely driven by the active vasculitis. However, given the patient’s pre-existing autoimmune disease, immunosuppressant therapy, and the weeping nature of the blistering skin lesions, infection should always be considered and treated empirically.

In this case, the patient was initially treated for infection while awaiting further investigations, including a skin biopsy and MRI of the orbits, before immunosuppressive treatment was started. Could the vision loss have been prevented if treatment had been commenced sooner? A further review by ophthalmology revealed vision in the left eye had improved but the right eye was unchanged and has required ongoing review. This case demonstrated the complexities with management of a patient where infectious and inflammatory causes were simultaneously in question.

Furthermore, the importance of recognising leukocytoclastic vasculitis and its possible skin manifestations, including bullous lesions, is highlighted by this case. Cutaneous vasculitis should be considered in patients with this specific skin morphology, regardless of previous negative skin biopsies.

